# Liquid biopsy in biliary tract cancer from blood and bile samples: current knowledge and future perspectives

**DOI:** 10.37349/etat.2022.00087

**Published:** 2022-06-22

**Authors:** Gianluca Arrichiello, Valeria Nacca, Fernando Paragliola, Emilio Francesco Giunta

**Affiliations:** 1 Oncology Unit, Department of Precision Medicine, Università degli Studi della Campania Luigi Vanvitelli, 80131 Naples, Italy; 2 Department of Experimental Medicine, Università degli Studi della Campania Luigi Vanvitelli, 80138 Naples, Italy; Humanitas University, Humanitas Research Hospital, Italy

**Keywords:** Biliary tract cancer, liquid biopsy, plasma, bile, tumor biomarkers

## Abstract

Biliary tract cancer (BTC) is an aggressive tumor characterized by a poor prognosis. In the latest years, targetable genetic alterations have been discovered in BTC patients, leading to the approval of new targeted therapies. Liquid biopsy, which is a non-invasive method for detecting tumor biomarkers from fluid samples, is a useful tool for diagnosis and molecular characterization, but also for prognosis assessment and monitoring of treatment response. In this review, recent works on liquid biopsy in BTC patients were analyzed, focusing on some relevant aspects for clinical use and trying to depict the future role of this technique. Moreover, differences between plasma and bile samples were pointed out, in light of the peculiar biology of BTC and the possibility of using bile as an alternative source of cell-free DNA (cfDNA) for genomic analysis. In the era of precision oncology, the increasing adoption of liquid biopsy in BTC patients will certainly improve the management of this disease.

## Introduction

### Epidemiology of biliary tract cancer

Biliary tract cancer (BTC) encompasses a heterogeneous group of tumors that differ on the basis of etiology, natural history, morphology, and molecular alterations. Depending on their anatomical location, BTCs are subclassified as intrahepatic cholangiocarcinoma (iCCA), originating from the biliary tree within the liver, or extrahepatic cholangiocarcinoma (eCCA) outside the liver parenchyma; eCCA is further subdivided into perihilar cholangiocarcinoma (pCCA or Klatskin tumor) and distal cholangiocarcinoma (dCCA) [1, 2]. The two most common forms are pCCA (50–60%) and dCCA (30–40%) with iCCA representing only 10–20% of the total. In addition, BTC also includes gallbladder cancer (GBC) [3, 4].

Cholangiocarcinoma (CCA) can arise from epithelial cells in the biliary surface epithelium (i.e. cholangiocytes) and in peribiliary glands; evidence has accumulated, suggesting that iCCA might also originate from mature hepatocytes [5–7]. The most frequently reported precursor lesions include biliary intra-epithelial neoplasia and intraductal papillary neoplasms of the bile duct [8].

The incidence of CCA is definitely lower in Europe, the USA, and Australia (0.3–3.5/100,000), but higher in the areas of the world where liver fluke infections are widespread (Thailand, China, and Korea) [9]; similarly, the incidence of GBC is low in Western Europe and USA (1.6–2.0/100,000) whereas, in Chile, India, and Eastern Europe is an important health problem. BTC occurs mostly in the seventh decade and mainly in the male sex (male:female ratio of 1.2–1.5:1.0) [10].

Historically CCA is an aggressive cancer, and it is associated with a poor prognosis for several factors, i.e. the advanced stage at diagnosis, for only one-third of patients can undergo surgery which is potentially curative, but also the limited systemic treatment options available. For patients with unresectable or advanced CCA, the standard first-line systemic therapy consists of a combination of cisplatin and gemcitabine, which yields a median overall survival (OS) of 11.7 months, as shown in the phase III trial by Valle et al. [11]. Recently, in the second-line setting, the ABC-06 trial showed that the FOLFOX regimen was associated with a marginal improvement in median OS compared with best supportive care (6.2 months *versus* 5.3 months) [12].

With such limited benefits and a survival rate of less than 10% at 5 years [4], there is the need for a better characterization of the disease; this has become possible by understanding the genetic alterations acting as oncogenic drivers in CCA, which could therefore be targeted by specific therapies.

### Genomic alterations in BTC

Several studies have focused their attention on the molecular biology of BTC, highlighting different genetic alterations through whole-exome sequencing (WES) or focused sequencing—restricted to specific gene sequences.

The most commonly found alterations in iCCA are isocitrate dehydrogenase 1/2 (*IDH1/2*) mutations, fibroblast growth factor receptor 2 (*FGFR2*) fusions, B-Raf proto-oncogene (*BRAF*) and AT-rich interaction domain 1A (*ARID1A*) mutations, while in eCCA tumor protein p53 (*TP53*), SMAD family member 4 (*SMAD4*) and KRAS proto-oncogene (*KRAS*) mutations are more frequent [13]. The study conducted by Javle et al. [14] involving 554 CCA patients demonstrated that the presence of *FGFR2* fusions was associated with a better prognosis, while *TP53* and *KRAS* mutations were associated with a poor outcome. The frequency and type of genomic alterations described in this study are summarized in Table 1.

**Table 1.** Most frequently altered genes in BTCs

**Gene**	**Type of alteration**	**Frequency**	**References**
*IDH*	Mutation	6–36%	[13]
*FGFR*	Fusion	8–14%	[13–15]
*TP53*	Substitution/indel	27–59%	[13, 14]
*ARID1A*	Mutation	7–36%	[13]
*KRAS*	Substitution/indel	12–42%	[13, 14]
*SMAD4*	Mutation	21–30%	[13]
*CDKN2A*	Deletion	19%	[13]

*CDKN2A*: cyclin dependent kinase inhibitor 2A; indel: insertion-deletion mutation

The most relevant actionable gene alterations in CCAs are *FGFR2* fusions and IDH1 mutations. In view of results from the FIGHT-202 phase II clinical trial, pemigatinib—a selective FGFR1–3 inhibitor—received regulatory approval as a second-line treatment option in locally advanced or metastatic CCA with FGFR2 fusions or rearrangements [15].

Analogously, in the ClarIDHy phase III clinical trial, the IDH1 inhibitor ivosidenib provided significant progression-free survival (PFS) benefit when compared to placebo in refractory IDH1 mutant BTC patients (median: 2.7 months *versus* 1.4 months) [16].

Therefore, it appears essential to evaluate genomic characteristics to guide treatment decisions; this has been made easier nowadays thanks to several methods such as polymerase chain reaction (PCR) and, most recently, next-generation sequencing (NGS) platforms, which allow to get parallel sequencing of a large number of genes [17].

NGS enables the detection of a wide variety of genetic alterations, including single-nucleotide polymorphisms, mutations, insertions, and deletions with high accuracy.

Genomic tests are usually performed on formalin-fixed paraffin-embedded (FFPE) tumor tissue; however, difficulties in obtaining an adequate sample size could hamper molecular evaluation in BTC. Therefore, liquid biopsy provides a window of opportunity to acquire information otherwise not easily available [18].

Liquid biopsy is a non-invasive and safe method that can be useful in the clinical diagnosis and treatment management in patients with cancer: in fact, it comprises different methodologies aimed at detecting tumor biomarkers from fluid samples such as blood, urine, plasma, and bile. In particular, this methodology allows the study of DNA, RNA, microRNA (miRNA) and proteins, derived from the primary or metastatic tumor, whether they are free or contained in circulating tumor cells (CTC), extracellular vesicles or exosomes [19]. It has also the advantage to provide a real-time picture of the tumor within a single patient, since it can be used for early diagnosis or identification of minimal residual disease, but also to monitor the response to therapy and the genetic evolution of the neoplasm; in addition, it offers the possibility of evaluating tumor heterogeneity, identifying potential molecularly targeted therapies and emerging mechanisms of resistance to chemotherapy [20].

Liquid biopsy on plasma sample, compared to traditional techniques for the diagnosis of cancer, has several advantages: it is a minimally invasive test with few complications (there is no risk of hemorrhage, infection, or tumor seeding); moreover, the venous blood sample can be taken in any health facility [21]. However, the high costs of analysis are, to date, a limit for the spread of this approach.

In the following paragraphs, we will explore the most recent discoveries concerning liquid biopsy in BTC patients and analyze current and upcoming applications of this technique, focusing on efforts made on both peripheral blood and bile, whose exploitation is peculiar to this kind of disease.

### Methods

We reviewed the available literature on liquid biopsy in BTC. We performed PubMed and Embase searches focused on blood and bile-based liquid biopsy applications, selecting primary and review articles from peer-reviewed journals. Search terms included “biliary tract cancer”, “liquid biopsy”, “bile-based biopsy”, “circulating tumor DNA”, and “microRNA”. We also searched PubMed and major oncology conferences for relevant presentations on this topic and browsed ClinicalTrials.gov to find ongoing clinical trials pertinent to the matter of this review.

## Blood-based biopsy in BTC

Blood-based liquid biopsy has several potential applications in BTC management, such as facilitating diagnosis as an adjunctive method to traditional investigations (if they are unfeasible or non-diagnostic), risk stratification and prognosis evaluation, development of precision medicine strategies, detection of relapse, and emerging resistance mechanisms [22].

Expectedly, considering the ease of sample collection, most of the efforts made in the liquid biopsy field have involved blood-based biopsies. Work produced so far focused on the analysis of cell-free DNA (cfDNA), circulating tumor DNA (ctDNA)—released in the circulation after metabolic secretion or cellular death—exosomes, miRNAs, serum proteins, cytokines and metabolites, and CTCs [23].

In this section we describe the most recent and relevant research made in this field, focusing on the role of blood-based liquid biopsy in diagnosis and molecular characterization of BTC, risk evaluation and prognosis assessment, and monitoring of response to treatment. Since most of the research has been produced on either cfDNA or ctDNA, we will focus on this setting as well.

### Diagnosis and molecular characterization

Commercial availability of several assays to process plasma and extract genomic material has allowed the detection of genetic and epigenetic alterations associated with BTC and improved molecular characterization of this disease.

Kumari et al. [24] assessed the role of cfDNA in the diagnosis of GBC using samples collected from 34 patients and 39 matched controls, which included individuals affected by cholecystitis. The cfDNA level was significantly lower in controls compared to patients, and higher levels correlated with stage and burden of disease.

Following this application, Wasenang et al. [25] developed a different approach to discriminate between CCA and non-tumoral biliary disease (i.e. chronic cholecystitis, cholangitis, cholelithiasis) relying on the methylation level of opioid binding protein cell adhesion molecule-like (*OPCML*) and 5’-Hoxd genes (*HOXD9*) as determined in cfDNA; the combined assessment showed high specificity and positive predictive value, suggesting that it could prevent misdiagnosis of CCA, thus potentially sparing unnecessary surgical procedures.

Molecular profiling is becoming increasingly relevant in the management of patients with advanced CCA. Mody et al. [26] reported results from the largest cohort to date in Western patients, analyzing 138 samples from a series of 124 patients with advanced CCA to characterize the ctDNA genomic alteration landscape in patients with BTCs; the majority of patients had intrahepatic tumors and most of the samples were processed with a 73-gene panel, yielding one or more alterations in 76% of patients. Clinically relevant alterations were observed in approximately half the patients, including *BRAF* mutations, erb-b2 receptor tyrosine kinase 2 (*ERBB2*) amplifications, *FGFR2* fusions, *FGFR2* mutations, and *IDH1* mutations. Interestingly, a different array of alterations was observed in patients younger than 50 years compared to older ones, although overall the differences were not statistically significant.

Blood-based assays feature high concordance with tumor tissue biopsies. Zill et al. [27] managed to prospectively analyze 54 genes in both tumor and cfDNA, demonstrating a concordance of 90%. Similarly, Andersen and Jakobsen [28] tested a multiplex digital PCR method to search for mutations in *KRAS*, NRAS proto-oncogene (*NRAS*), *BRAF*, and phosphatidylinositol-4,5-bisphosphate 3-kinase catalytic subunit alpha (*PIK3CA*) genes in the plasma of 5 CCA patients with known tumor tissue mutations and found that the mutations present in the tumor were also detected in plasma samples in 100% of cases.

Further on this, Lamarca et al. [29] recently reported results from the analysis of 112 paired tissue and plasma samples from 104 individual patients; focusing on the blood-based analysis, identification of pathological findings was possible regardless of whether the patient was on active treatment or not. Notably, also in this work, concordance of findings for paired tissue-ctDNA was complete. Similar work was conducted on Asian patients by Chen et al. [30] who recently published the results of an NGS-based analysis adopting a 150-gene panel in 154 advanced CCA Chinese patients. Ninety-four point eight percent of patients had at least one genetic alteration detected in their ctDNA, with *KRAS* and *TP53* being the most frequently mutated genes; concordance with tumor tissue analysis was in line with previously published works.

Liquid biopsy can also be informative beyond the mere research of genetic alterations. A recent work produced by Csoma et al. [31] found a positive correlation between cfDNA yield and the estimated tumor volume; when comparing liquid biopsy results to those from tissue analysis, a similar tumor variant burden (TVB) was reported and some of the most relevant aberrations in BTC—such as those involving *FGFR2*, *IDH1*, *IDH2*, *KRAS*, and *TP53*—could be detected in both samples; interestingly, in 2 cases, only the serum sample had been informative for molecular assessment.

Moreover, Okamura et al. [32] analyzed a cohort of 121 patients who reported a higher concordance when matching ctDNA-metastatic tumor tissue than ctDNA-primary tumor tissue.

### Risk evaluation and prognosis assessment

A blood-based liquid biopsy might also allow to improve the definition of patient prognosis and tailor treatment accordingly.

Ettrich et al. [33] collected tumor tissue and corresponding ctDNA samples from 24 patients with either intrahepatic or extrahepatic BTC before and during treatment and proceeded to deep sequencing of a panel of frequently mutated genes. Blood/tissue concordance in this set was lower (74%) than previously reported—although higher in iCCA alone—but, interestingly, ctDNA detection of mutations in BReast CAncer gene 1 (*BRCA1*) associated protein 1 (*BAP1*), polybromo 1 (*PBRM1*), *KRAS* was shown to predict a shorter PFS; these results support the role of liquid biopsy in estimating the prognosis of patients with BTC.

On the other hand, the already cited work by Lamarca et al. [29] found no correlation between either PFS and OS and median maximum mutated allele frequency (MAF), evaluated on ctDNA samples taken before starting therapy.

Interestingly, the same working group evaluated a small cohort of 11 patients with pancreato-biliary malignancies to determine the role of ctDNA analysis in detecting minimal residual disease after potentially curative surgical resection [34]. Although not statistically significant, there was indeed a trend towards increased relapse risk in the patients with ctDNA present following surgery.

### Precision medicine applications

More accessible and precise molecular characterization allows for broader and easier use of targeted therapy. A blood-based liquid biopsy might facilitate the monitoring of response to treatment, as well as detection and targeting of emerging mechanisms of treatment resistance.

A retrospective analysis from the aforementioned ClarIDHy trial—which evaluated the efficacy of IDH1 inhibitor ivosidenib in mutant *IDH1* CCA—showed that *IDH1* mutation clearance as evaluated on plasma ctDNA in patients followed longitudinally correlated with disease control [35]; data from the same cohort also confirmed the feasibility of *IDH1* mutation detection in plasma, with a blood-tissue concordance of 92%.

The group of Yang et al. [36] recently used a model based on the copy number variations (CNVs) detected in 187 hepatobiliary cancer patients to predict the benefit of combination immune checkpoint inhibitor (ICI) therapy; published results showed that a low CNV risk score was associated with longer OS and PFS.

Furthermore, the aforementioned study conducted by Okamura et al. [32] evaluated the outcome of 34 BTC patients treated with a personalized approach based on molecular characterization (via either liquid or tissue biopsy), and compared to the outcome of 46 BTC patients treated with unmatched regimens; both response rate (RR) and PFS were significantly greater in the matched treatment group than the unmatched treatment group [hazard ratio (HR) = 0.60, 95% confidence interval (CI) = 0.37–0.99, *P* = 0.047] [32].

Anecdotally, Yarlagadda et al. [37] reported a successful attempt at personalized treatment in advanced CCA harboring *ERBB2* amplification, as detected by ctDNA analysis; the patient was treated with a combination of anti-HER2 drugs, trastuzumab, and pertuzumab, and achieved rapid and sustained clinical benefit. Interestingly, after just one administration, the dominant *TP53* mutation in ctDNA reduced from 60.7% to 2.1%, and the plasma copy number of *ERBB2* declined as well.

Similarly, Yu et al. [38] recently identified a novel EH domain-binding protein 1-MET proto-oncogene receptor tyrosine kinase (*EHBP1-MET*) fusion by performing NGS analysis on both tumor tissue and ctDNA from a 41-year-old patient with iCCA; the patient was subsequently treated with MET inhibitor crizotinib and managed to achieve durable tumor response.

The previously mentioned work by Ettrich et al. [33] showed also that 63% of patients showed a change in their mutational profile during treatment, with variants in a subset of genes possibly driving tumor progression. Moreover, serial analysis of ctDNA in *FGFR2* fusion-positive CCA patients treated with FGFR inhibitor ingrafitinib demonstrated multiple recurrent point mutations in the FGFR2 kinase domain at progression [39]. Interestingly, subsequent *in vitro* studies indicated that each mutation was surmountable by structurally distinct FGFR inhibitors, thus suggesting that this approach might lead to the development of individually targeted therapeutic strategies.

The same authors reported that TAS-120, an irreversible pan-FGFR inhibitor, was effective in 4 patients with FGFR2 fusion-positive CCA who developed resistance to previous FGFR inhibition and were selected through examination of serial biopsies, ctDNA, and patient-derived tumor cells [40] (Table 2).

**Table 2.** Blood-based liquid biopsy in BTCs

**Authors**	**Number of patients**	**Type of analysis**	**Relevance**	**References**
Kumari et al.	73	cfDNA/qPCR	Higher cfDNA levels aid differential diagnosis with benign biliary conditions and correlate with the burden of disease	[24]
Wasenang et al.	80	cfDNA/MS-HRM	Levels of methylation of *OPCML* and *HOXD9* can help differential diagnosis with benign biliary conditions	[25]
Mody et al.	138	ctDNA/Guardant^®^	Blood-based liquid biopsy can be used for molecular characterization and can identify clinically relevant alterations	[26]
Zill et al.	26	cfDNA/NGS	Blood-based liquid biopsy features a high level of concordance with tumor tissue analysis	[27]
Andersen and Jakobsen	11	ctDNA/multiplex digital PCR	Blood-based liquid biopsy features a high level of concordance with tumor tissue analysis	[28]
Lamarca et al.	104	ctDNA/FoundationOne Liquid^®^	Blood-based liquid biopsy features a high level of concordance with tumor tissue analysis	[29]
Chen et al.	154	ctDNA/NGS	Blood-based liquid biopsy features a high level of concordance with tumor tissue analysis and can identify genomic alterations in the majority of patients tested	[30]
Csoma et al.	25	cfDNA/NGS	cfDNA yield correlates with tumor burden; blood-based biopsy can be informative beyond tumor tissue analysis	[31]
Okamura et al.	121	ctDNA/Guardant^®^	Blood-based liquid biopsy features a higher concordance with metastatic tumor tissue than with primary tumor tissue	[32]
Ettrich et al.	24	ctDNA/NGS	Detection of mutations in *BAP1, PBRM1,* and *KRAS* via blood-based liquid biopsy was shown to predict a shorter PFS	[33]
Lamarca et al.	11	ctDNA/FoundationOne Liquid^®^	The presence of ctDNA following surgery could predict increased relapse risk	[34]
Aguado et al.	210	ctDNA/digital PCR	Blood-based liquid biopsy can identify *IDH1* mutations and select patients for targeted therapies	[35]
Yang et al.	187	cfDNA/NGS	CNV detection by liquid biopsy can predict response to immunotherapy	[36]
Yarlagadda et al.	1	ctDNA/Guardant^®^	Blood-based liquid biopsy can identify uncommon targetable genomic alterations	[37]
Yu et al.	1	ctDNA/NGS	Blood-based liquid biopsy can identify uncommon targetable genomic alterations	[38]
Goyal et al.	3	ctDNA/ddPCR, Guardant^®^	Blood-based liquid biopsy can identify mechanisms of resistance to treatment	[39]
Goyal et al.	4	ctDNA/ddPCR, Guardant^®^	Blood-based liquid biopsy can help overcome resistance to targeted therapies	[40]

ddPCR: droplet digital PCR; qPCR: quantitative PCR; MS-HRM: methylation-sensitive high-resolution melting

## Bile based liquid biopsy in BTC

Biological fluids other than blood, such as urine, cerebrospinal fluid, and bile can be used to recover cfDNA, ctDNA, circulating RNA, and miRNAs [41].

Bile, in particular, might represent a potential alternative to either tissue or plasma for the diagnosis and the evaluation of prognostic and predictive biomarkers [42, 43]. Endoscopic or percutaneous biopsy for bile collection is undoubtedly invasive but, in BTC, bile is in direct contact with tumor cells; considering tumor heterogeneity, bile could theoretically provide more accurate information than a tissue biopsy.

Given these premises, several studies have evaluated the performance of biliary cfDNA analysis over both plasma and tissue analysis.

Early work by Shi et al. [44] assessed the possibility of detecting *KRAS2* point mutations in biliary samples drawn from patients with a variety of neoplastic and non-neoplastic pancreato-biliary diseases. Interestingly, the mutation levels were significantly lower in the non-neoplastic bile (median = 0.4%) compared with those in the neoplastic bile (median = 5.1%).

In the study by Shen et al. [45], bile samples from 10 BTC patients were analyzed through electropherograms—for measuring cfDNA length—and deep sequencing—for identifying mutations on the panel of 150 tumor-related genes; cfDNA fragments were 6,000 base pairs (bps) long, which is higher than cfDNA isolated from plasma, and a concordance of mutations between tumor tissue and bile was found in 9 out of 10 patients. Among the most frequent mutated genes, there were *TP53*, *KRAS*, and neurogenic locus notch homolog protein 1/2 (*NOTCH1/2*).

In another study conducted by Gou et al. [46], based on 28 patients with BTC, authors analyzed the genomic mutational profile of tumoral tissue DNA, plasma cfDNA, and biliary cfDNA using a panel of 520 genes related to BTC; intriguingly, biliary cfDNA was found to be superior to plasma cfDNA in detecting somatic mutations, showing a concordance of 71.4% and 53.6% with tumor tissue, respectively. In this study, the most frequent mutated genes found in bile were *TP53*, *CDKN2A* and *KRAS*.

In the study conducted by Driescher et al. [47], conducted on 45 patients affected by pancreatic ductal adenocarcinoma (*n* = 35) and CCA (*n* = 10), bile-based biopsy performed better than plasma-based biopsy; in fact, using a panel-based NGS, the concordance bile-tissue and plasma-tissue was 96.2% and 31.6%, respectively. Comparing results from the same patients, less than 50% of mutations found in bile samples were also present in plasma samples.

In line with these results, in the study conducted by Han et al. [48] in 42 BTC patients, bile cfDNA showed superior mutational concordance to matched FFPE tumor tissue than plasma tissue (80% *versus* 42.9%, respectively). Authors have also evaluated the prognostic role of *KRAS* mutation in bile samples, showing a worse survival in patients with the detectable mutation.

Liquid biopsy on bile samples is a valid tool for the analysis of biomarkers useful for therapeutic planning and treatment monitoring, but it is also helpful for early diagnosis. Arechederra et al. [49] evaluated the use of a panel-based NGS named “Bilemut” in a prospective cohort of 68 patients with suspicious biliary strictures, reaching a sensitivity and specificity for malignancy of 96.4% and 69.2%, respectively. In the aforementioned work by Gou et al. [46], bile cfDNA was able to increase the sensitivity of the serum marker carbohydrate antigen 19-9 (CA19-9), which achieved 96.4% in BTC diagnosis, higher than the sensitivity of CA19-9 combined with plasma cfDNA obtained in the same population (Table 3).

**Table 3.** Bile-based liquid biopsy in BTCs

**Authors**	**Number of patients**	**Type of analysis on bile cfDNA**	**Relevance**	**References**
Shen et al.	10	Targeted deep sequencing	Bile-based liquid biopsy features high concordance with blood samples and tumor tissue	[45]
Gou et al.	28	qPCR	Bile-based liquid biopsy features high concordance with blood samples and tumor tissue	[46]
Driescher et al.	35	NGS (qPCR for KRAS analysis)	Bile-based liquid biopsy features high concordance with blood samples and tumor tissue and can outperform blood-based biopsy	[47]
Han et al.	42	qPCR	Detection of *KRAS* mutations in bile samples predicts poorer prognosis	[48]

## Future perspectives

Liquid biopsy, in its simplicity of execution, represents a feasible alternative to tissue biopsy for both diagnosis and molecular characterization, all of which are currently required for clinical trial enrollment.

In this context, we report the ongoing observational prospective trial NCT04445532 (acquisition of blood and tumor tissue samples from patients with hepatobiliary tumors); this trial is designed to identify biomarkers of response by analyzing different samples—including plasma and bile—and survival in both resectable and advanced hepatobiliary tumors and to contribute to the molecular characterization of this disease.

The use of liquid biopsy in BTC patients will certainly modify the way these patients are monitored during therapies, as it is already happening in other tumor types; an ongoing clinical trial is evaluating the role of blood sampling to correlate cfDNA and CTC analysis to response and progression on chemotherapy in BTC patients receiving gemcitabine/cisplatin/nab-paclitaxel chemotherapy, by collecting samples at specific timepoints (NCT04871321).

Most of the recent efforts in liquid biopsy application in BTC have focused on miRNA, so we will dedicate the rest of this section to describe emerging evidence regarding their role as diagnostic, prognostic, and predictive biomarkers.

The need for biomarkers to improve diagnosis, prognosis, and predictiveness of treatment efficacy, has led to the investigation of the role of miRNAs [50], which play an important role in neoplastic progression and metastasis by regulating processes of cell differentiation, proliferation, and apoptosis, as well as angiogenesis and epithelial-mesenchymal transition [51, 52].

miRNAs can be detected in body fluids such as plasma and bile following exocytosis, apoptosis, and other cellular processes; as such, miRNAs can be used as diagnostic, prognostic, and predictive biomarkers of sensitivity and resistance to therapy.

miRNAs can be of help in the early and differential diagnosis of BTC. miRNA-21 behaves as an oncogenic miRNA by directly targeting the expression of tumor suppressor genes phosphatase and tensin homolog (*PTEN*) and protein tyrosine phosphatase non-receptor type 14 (*PTPN14*) [53].

Another miRNA of interest is miR-150, which acts as a tumor suppressor since the knockdown of its expression induces tumor cell proliferation, migration, and invasion *in vitro* [54]. Wu et al. [55] used samples of blood and bile from a cohort encompassing healthy controls, patients with primary sclerotizing cholangitis (PSC), and CCA and found that miR-150-5p level was consistently lower in CCA samples than in normal control and PSC; moreover, miR-150-5p levels were associated with serum CA19-9 levels and CCA pathological grade.

Voigtländer et al. [56] analyzed 83 bile samples from patients with BTC and PSC, highlighting the down-regulation of 10 miRNAs in patients with cancer compared to benign disease.

Han et al. [57] analyzed biliary samples from 106 patients with obstructive biliary disease, either benign or tumoral, and found that miR-30d-5p and miR-92a-3p were significantly upregulated in patients with CCA. Authors also suggested said miRNAs might participate in CCA-associated signaling pathways such as Hippo, Wnt, p53, mitogen-activated protein kinase (MAPK), and EGFR [57].

The prognostic and predictive role of miRNAs, analyzed on both blood and bile samples, have been studied in BTC patients. MiR-106, for example, has been associated with the onset of lymph node metastases after surgical resection and with reduced survival, while miR-26 has been associated with reduced OS and reduced disease-free survival [58, 59].

Meng et al. [60] highlighted the association between the inhibition of miR200b and miR-21 and the increased sensitivity to gemcitabine, with an increased cytotoxic effect. The downregulation of let-7a by the inflammatory cytokine interleukin-6 (IL-6) has also been associated with an increased apoptotic effect during treatment with gemcitabine, 5-fluorouracil, or irinotecan [61]. Okamoto et al. [62] demonstrated a correlation between gemcitabine resistance and increased expression of miR-125-5p as well as reduced expression of miR-221, 29b, and 205: these three miRNAs, when up-regulated, promote sensitivity to gemcitabine; vice versa, when down-regulated, they cause resistance to this chemotherapeutic agent.

## Conclusions

The prognosis of BTC patients has dramatically improved in the latest years thanks to a better understanding of biology and the consequent development of new drugs targeting specific molecular alterations. Liquid biopsy has certainly provided new possibilities in managing BTC patients, from molecular diagnosis to prognosis assessment. However, the results produced so far have limited applications and may be inconsistent due to the small sample size. Therefore, more efforts are needed to incorporate its use in clinical practice, taking in mind that high costs are the greatest limit in its widespread application.

The systematic adoption of liquid biopsy in the design of future BTC clinical trials is highly recommended to clarify when and how to use it, especially regarding the monitoring of response and the identification of molecular mechanisms responsible for treatment resistance.

## Abbreviations

BRAF: B-Raf proto-oncogene
BTC: biliary tract cancer
CA19-9: carbohydrate antigen 19-9
CCA: cholangiocarcinoma
cfDNA: cell-free DNA
CNVs: copy number variations
ctDNA: circulating tumor DNA
eCCA: extrahepatic cholangiocarcinoma
*ERBB2*: erb-b2 receptor tyrosine kinase 2
FGFR2: fibroblast growth factor receptor 2
GBC: gallbladder cancer
iCCA: intrahepatic cholangiocarcinoma
IDH1/2: isocitrate dehydrogenase 1/2
KRAS: KRAS proto-oncogene
miRNA: microRNA
NGS: next-generation sequencing
OS: overall survival
PCR: polymerase chain reaction
PFS: progression-free survival
PSC: primary sclerotizing cholangitis
TP53: tumor protein p53

## Declarations

### Copyright

© The Author(s) 2022.
